# A new approach to streamline obstructive sleep apnea therapy access using peripheral arterial tone-based home sleep test devices

**DOI:** 10.3389/frsle.2023.1256078

**Published:** 2023-11-06

**Authors:** Ding Zou, Steven Vits, Carlos Egea, Daniela Ehrsam-Tosi, Florent Lavergne, Mikel Azpiazu, Ingo Fietze

**Affiliations:** ^1^Center for Sleep and Vigilance Disorders, Institute of Medicine, Sahlgrenska Academy, University of Gothenburg, Gothenburg, Sweden; ^2^Faculty of Medicine and Health Sciences, University of Antwerp, Antwerp, Belgium; ^3^EctoSense, Leuven, Belgium; ^4^Respiratory Service and Sleep Unit, Araba University Hospital, Bioaraba Scientific Institute, Ciberes, Vitoria-Gasteiz, Spain; ^5^ResMed Science Center, Basel, Switzerland; ^6^ResMed Science Center, Saint-Priest, France; ^7^Bioaraba, Neurosciences-Sleep Disorders, Osakidetza Basque Health Service, Araba University Hospital, Sleep Unit, Vitoria-Gasteiz, Spain; ^8^Interdisciplinary Center of Sleep Medicine, Charité-Universitätsmedizin Berlin, Berlin, Germany

**Keywords:** diagnosis, peripheral artery tonometry, precision medicine, sleep disordered breathing, telehealth, HSAT, night-to-night variability

## Abstract

Obstructive sleep apnea (OSA) is a prevalent condition that negatively impacts cardiovascular, metabolic and mental health. A high proportion of individuals with OSA remain undiagnosed and incur significant healthcare costs. The gold standard OSA diagnostic is in-lab polysomnography, but this is costly and time-consuming. Home sleep apnea tests (HSATs), including cardiorespiratory polygraphy and peripheral artery tonometry technology, provide an alternative. Advances in HSAT technology include non-invasive, easy-to-use medical devices that could allow unobtrusive, accessible, multi-night, cost-effective diagnosis and management of sleep-disordered breathing. One type of these devices is based on determination of peripheral arterial tone, and use photoplethysmography signals from the finger (oxygen saturation, pulse wave amplitude and pulse rate). The devices contain algorithms that use these data to generate the traditional metrics required by the American Academy of Sleep Medicine. They can be used to record sleep parameters over multiple nights at home, and can also provide information on total sleep time (TST) and sleep stages (including time spent in rapid eye movement sleep). The combination of objective measures (apnea-hypopnea index, oxygen desaturation index, respiratory disturbance index, TST) and subjective measures (symptoms and other patient-reported outcome measures) could facilitate the development of a personalized therapeutic plan for OSA patients. It is anticipated that the streamlined digital pathway facilitated by new peripheral artery tone-based technology could contribute to reducing the underdiagnosis of OSA, accelerating access to appropriate treatment, and the optimization of OSA therapy.

## 1. Introduction

Obstructive sleep apnea (OSA) is the most common form of sleep-disordered breathing (SDB). It is characterized by partial or complete upper airway obstructions that are associated with intermittent hypoxia and transient arousals. The global prevalence of OSA in middle-aged adults has been estimated to be nearly one billion, with approximately half of these having moderate-to-severe disease with an indication for treatment (Benjafield et al., 2019).

OSA results in increased sympathetic activity, oxidative stress, inflammation, endothelial and metabolic dysfunction, and is associated with a variety of cardiovascular, cerebrovascular and metabolic diseases, and increased all-cause mortality (Nieto et al., 2000; Peppard et al., 2000; Kendzerska et al., 2014; Kent et al., 2015; Linz et al., 2015; Reutrakul and Mokhlesi, 2017; Xie et al., 2017; Mehra et al., 2022; Salari et al., 2022). Untreated OSA also contributes to occupational and traffic accidents (Bioulac et al., 2017; Hirsch Allen et al., 2020) and absence from work (Lallukka et al., 2014), and has a negative impact on cognitive function (Gnoni et al., 2023) and quality of life (Kerner and Roose, 2016; Vinnikov et al., 2017; Alomri et al., 2021; Legault et al., 2021).

A high proportion of individuals with OSA remain undiagnosed (Young et al., 1997; Kapur et al., 2002). This is relevant from a health system perspective because a person with OSA has been estimated to have double the annual healthcare costs than someone without OSA (Kapur et al., 1999). Furthermore, the diagnosis and treatment of OSA are associated with positive economic benefit (Wickwire, 2021; Mattila et al., 2022; Sterling et al., 2023).

## 2. OSA diagnosis

The current gold standard for diagnosing OSA is in-laboratory polysomnography (PSG). PSG is a costly and time-consuming process that requires highly trained personnel for set-up and scoring, and therefore has limited availability. PSG is essential in specific patient groups (e.g., those with comorbidities), but the majority of individuals do not require PSG for diagnosis of OSA. PSG is subject to the first-night effect and although it can be performed over multiple nights and at home, this is resource intensive and not feasible in the majority of cases, which limits its ability to detect night-to-night variability in SDB parameters (Newell et al., 2012). Therefore, there is a need for OSA diagnostic tests that are more widely available, cost effective and can be used for timely multi-night sleep testing, allowing healthcare professionals to take care of all individuals referred for evaluation or management of OSA. As a result, home sleep apnea testing (HSAT) has become a routine approach for individuals with suspected OSA. HSAT does not require supervision, is less expensive than PSG and allows replication of sleep patterns under “usual” conditions. Many PSG-validated HSAT devices are available (e.g., level 3 cardiorespiratory polygraphy) that provide adequate apnea-hypopnea index (AHI) estimation according to the American Academy of Sleep Medicine (AASM) criteria for sleep apnea diagnosis (Kapur et al., 2017; Rosen et al., 2018). However, use of total recording time rather than total sleep time (TST) to calculate respiratory indices may lead to important underestimation of event rates (Escourrou et al., 2015; Massie et al., 2022a). According to the AASM practical guidelines, both polygraphy and peripheral artery tonometry-based HSATs can be used for the diagnosis of sleep apnea (American Academy of Sleep Medicine, 2023). There are two Conformité Europénne (CE) mark and US Food and Drug Administration-approved, commercially available peripheral artery tonometry-based HSAT devices (NightOwl^®^ and WatchPAT^®^).

### 2.1. Photoplethysmography and peripheral arterial tonometry for detection of respiratory events

Reflectance-based photoplethysmography (PPG) detects pulsatile changes in blood volume in peripheral tissues and has been defined as an important technology in sleep monitoring devices (Ryals et al., 2023). Peripheral artery tonometry refers to the determination of peripheral arterial vascular tone (the net balance between vasoconstriction and vasodilation) using PPG data. Peripheral artery tonometry measures pulsatile volume changes in the digital vascular bed that are densely innervated (Schnall et al., 1999; Zou et al., 2004). In the context of OSA, there is increased sympathetic nervous system activity near the end of a respiratory event (obstructive apnea). The associated release of norepinephrine increases tone in the peripheral arteries, resulting in vasoconstriction and a reduction in the volume of blood displaced between systole and diastole. By measuring this relative change in blood volume, sudden changes in peripheral arterial tone that occur in response to respiratory events can be detected (O'Donnell et al., 2002). These pulse wave amplitude drops have been shown to be an important biomarker of cardiometabolic risk and outcomes (Hirotsu et al., 2020; Strassberger et al., 2021; Solelhac et al., 2023).

Peripheral artery tonometry-based devices combine information on changes in arterial volume with oxygen saturation (SpO_2_; both from the PPG signal) with data on peripheral arterial tone and heart rate (Yalamanchali et al., 2013; Massie et al., 2018; Van Pee et al., 2022; Lyne et al., 2023). During recording, a respiratory event is typically detected by analyzing the co-occurrence of one or more of the following events: oxygen desaturation; vasoconstriction (decreased peripheral artery tonometry signal); and increased pulse rate. Based on these data, peripheral artery tonometry devices contain proprietary algorithms that generate the traditional metrics required by the AASM Manual for the Scoring of Sleep and Associated Events (e.g., AHI, respiratory disturbance index) (American Academy of Sleep Medicine, 2023). The two currently available devices, NightOwl^®^ and WatchPAT^®^, have different proprietary algorithms and technical specifications. Both have been validated against PSG (Zou et al., 2006; Massie et al., 2018; Van Pee et al., 2022), and generally show good agreement with PSG for parameters such as the AHI and OSA severity (O'Brien et al., 2012; Yalamanchali et al., 2013; Camilon et al., 2014; Choi et al., 2018; Ioachimescu et al., 2020; Van Pee et al., 2022). Furthermore, information on sleep (e.g., TST, time spent in rapid eye movement [REM] sleep, wake time) can also be estimated using peripheral artery tonometry-based devices (Hedner et al., 2011; Massie et al., 2018; Zhang et al., 2020).

### 2.2. Detection of central sleep apnea and REM sleep using peripheral artery tonometry-based devices

Although OSA is the predominant sleep apnea subtype, central sleep apnea (CSA) is another important form of SDB (Dempsey, 2019). The mechanisms underlying these two types of sleep apnea are different, because CSA is characterized by a lack of respiratory drive, while OSA result from a partial or complete obstruction of the upper airways. In PSG, cessation of respiratory drive or effort can be inferred from the abdominal and thoracic respiratory effort belts. This information is not currently available from peripheral artery tonometry-based devices, but could be detected using fingertip PPG data. The fingertip PPG signal inherently contains respiratory information because blood flow to body extremities is influenced by alterations in thoracic pressure throughout the respiratory cycle (Ryals et al., 2023). Therefore, the PPG signal amplitude oscillates in synchrony with the respiratory cycle. This amplitude modulation can be isolated to retain a signal representing respiratory effort. The respiratory effort signal can then be used to classify respiratory events as being of an obstructive or central nature. Use of PPG has recently been shown to provide useful data for the detection of CSA in individuals with suspected sleep apnea (Sommermeyer et al., 2012; Massie et al., 2023).

Approximately 10%−36% of individuals with sleep apnea have REM-predominant OSA (Alzoubaidi and Mokhlesi, 2016), whereby SDB events are more pronounced during REM sleep (Varga and Mokhlesi, 2019). These individuals are at high risk for common OSA comorbidities, including atherosclerosis, hypertension, metabolic syndrome and diabetes (Mokhlesi et al., 2014; Acosta-Castro et al., 2018; Ljunggren et al., 2022). In order to properly define this phenotype, it is essential to be able to classify REM sleep with sufficient accuracy. Vasoconstrictions and oxygen desaturations detected in peripheral artery tonometry and SpO_2_ signal traces, respectively, show a different temporal pattern between REM and non-REM sleep (Lavie et al., 2000; Dvir et al., 2002; Herscovici et al., 2007; Choi et al., 2016). Furthermore, pulse rate low frequency power has been shown to increase in REM sleep (Chouchou and Desseilles, 2014). This means that PPG-based techniques can be used to detect REM sleep (Lavie et al., 2000; Zhang et al., 2020), although peripheral artery tonometry-based HSAT has lower sensitivity for REM detection than PSG (Massie et al., 2022b).

Overall, the ability of peripheral artery tonometry-based HSAT devices to detect REM sleep and their potential to differentiate between central and obstructive respiratory events increase the utility and application of these devices across a range of SDB types. They may also have clinical usefulness in individuals with comorbidities such as atrial fibrillation (Tauman et al., 2020; Jensen et al., 2023) and chronic obstructive pulmonary disease (Hansson et al., 2023).

### 2.3. Multi-night sleep testing

A key advantage of a peripheral artery tonometry-based approach is that it provides a convenient and low-cost option for multi-night testing. This is important because evaluating SDB over multiple nights provides a greater amount of data on respiratory parameters. This may help to achieve a correct diagnosis, and could allow evaluation of the evolution of sleep-related breathing disorders over time during the application of appropriate therapy. Peripheral artery tonometry devices are small, and therefore allow more natural (e.g., less supine) sleep due to the lack of cables compared with PSG. Furthermore, there is a large body of evidence showing that there is substantial night-to-night variation in sleep-related respiratory events, meaning that a single night of monitoring may be insufficient to allow reliable determination of sleep apnea severity at the individual level, resulting in misclassification in a substantial proportion of people (Punjabi et al., 2020; Roeder et al., 2020; Lechat et al., 2022). Furthermore, emerging evidence suggests that large night-to-night variability in sleep apnea severity (based on the AHI) is a predictor of uncontrolled hypertension (Lechat et al., 2023), and that sleep data from a single night of recording performed worse than multi-night testing with respect to cardiovascular risk prediction (Lechat et al., 2023). For data from multiple nights of sleep testing (at least three nights in total, including one night on the weekend), some experts believe that it is probably best to use the highest AHI value recorded to provide guidance regarding treatment initiation, rather than the average AHI. However, studies are needed to validate this approach. In summary, the multi-night monitoring capability of peripheral artery tonometry devices allows patient sleep trajectories over time to be determined in an accessible and acceptable manner, providing a clearer understanding of sleep habits and allowing better shared decision-making and more personalized therapy (Hrubos-Strøm et al., 2023; Lisik et al., 2023).

## 3. OSA diagnostic and management workflow using peripheral artery tonometry-based devices

**Figure 1:**
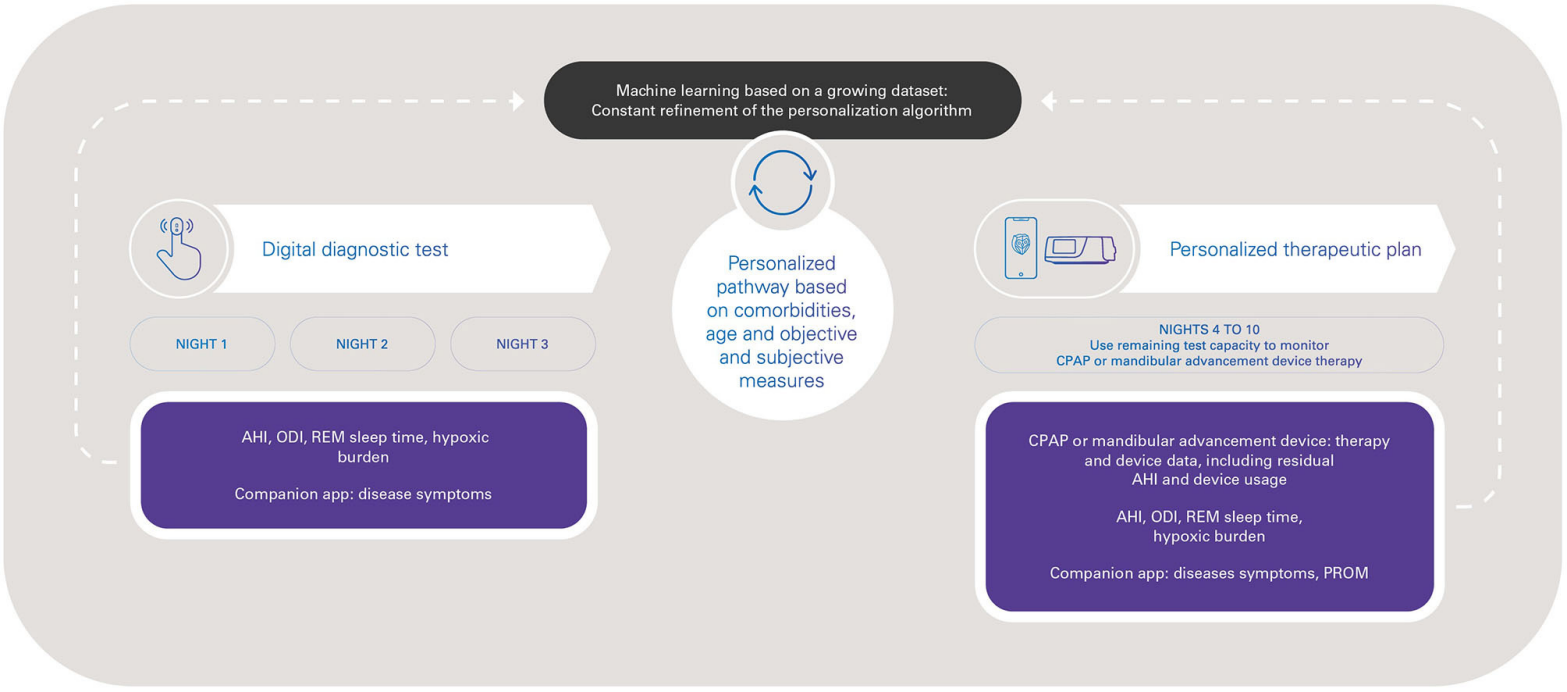
Toward a digital pathway for obstructive sleep apnea with peripheral arterial tone-based monitoring, from diagnosis to therapy management. AHI, apnea-hypopnea index; CPAP, continuous positive airway pressure; ODI, oxygen desaturation index; PROM, patient-reported outcome measures; REM, rapid eye movement.

HSAT workflow is simple and can be implemented remotely. However, a wider consideration is how new, multi-night, low-touch tools such as peripheral artery tonometry devices can be incorporated into the SDB patient pathway in a way that maximizes benefits for the patient (optimizing diagnosis and therapy), for healthcare professionals (time saving, reduced sleep lab workload, patient-centered management), and for the healthcare system (cost savings, resource optimization). As well as diagnosis, use of simple, compact HSAT devices could contribute to improving the efficiency of ongoing management of OSA therapies, including oral appliances and positive airway pressure therapies. An integrated and personalized diagnostic and therapeutic digital pathway can be facilitated by the use of objective diagnostic measures (AHI, oxygen desaturation index, sleep time, hypoxic burden), subjective measures such as symptoms and patient-reported outcome measures, and the monitoring of therapy efficacy (Figure 1).

For healthcare professionals, HSAT with a device that uses peripheral artery tonometry (such as NightOwl^®^ and WatchPAT^®^) is considered to be less time consuming, allowing more efficient patient management using a digital pathway without any loss of diagnostic accuracy. Having a solution that can be implemented remotely also allows more patients to be reached, especially those who do not have easy access to a sleep laboratory or sleep physician. In addition, the COVID-19 pandemic highlighted the value of being able to continue healthcare evaluations and treatment monitoring without face-to-face interaction between healthcare professionals and patients (Bouloukaki et al., 2023).

Accurate, multi-night sleep testing information is a key component that can help to drive much-needed personalized approaches to the diagnosis and treatment of sleep apnea (Arnardottir et al., 2022). While OSA may superficially be considered as a single disease, there are a variety of diverse clinical manifestations (or phenotypes) (Zinchuk et al., 2017; Duong-Quy et al., 2022). The presence of different OSA phenotypes means that a personalized, approach to the diagnosis and treatment of OSA is required to optimize clinical outcomes for individual patients (McNicholas and Korkalainen, 2023). The ability to detect different sleep apnea phenotypes such as REM-predominant OSA and CSA makes peripheral artery tonometry-based devices valuable tools for facilitating this type of personalized treatment.

Another important consideration is the patient experience, which is becoming increasingly recognized as a key measure of health system performance (Jamieson Gilmore et al., 2023). There are a number of features that would likely result in a good experience for individuals being investigated for SDB using peripheral artery tonometry-based devices. These include the ability to perform sleep testing over multiple nights in the home environment, simple device set-up, quick and reliable event analysis. This approach is also ideally suited to facilitate a P4 medicine approach to OSA—Predict; Prevent; Personalize; Participate (Lim et al., 2017). Early and effective diagnosis of OSA in otherwise healthy individuals would allow the implementation of lifestyle interventions and early treatment that could contribute to prevention of common OSA comorbidities (i.e., primary prevention) (Yim-Yeh et al., 2010), facilitate personalization of therapy options, and allow the individual to participate in the diagnosis and monitoring of their condition. Furthermore, simplicity and flexibility are important, especially for the disabled, the elderly and for people who are less familiar with new technologies.

## 4. Discussion

It has long been recognized that there is a lack of healthcare resources to meet the clinical demands of individuals with sleep apnea or suspected sleep apnea (Flemons et al., 2004; Pack, 2004). Nevertheless, effective and timely diagnosis of OSA plays an important role in preventing or limiting the negative health impacts of this condition. Peripheral artery tonometry-based wearable sleep testing devices that can be self-administered by the patient and scored automatically using validated artificial intelligence and machine learning-based algorithms have the potential to fill an important gap in healthcare service provision, improve access to diagnostic sleep studies and provide a cost-effective solution for sleep apnea diagnosis and monitoring.

Compared with conventional PSG, the benefits of peripheral artery tonometry-based wearable sleep testing devices include ease of performing evaluations over multiple nights. In addition, there will be savings in clinical staff time by avoiding complicated inventory, on-site desktop software updates, and cleaning and sterilization/disinfection procedures. However, these HSAT devices do not record a direct measurement of flow so it is not possible to distinguish between apneas and hypopneas (although both are counted), and there is no EEG-based sleep-staging (although information on sleep stages can be obtained by other means). Furthermore, there are some settings where use of peripheral artery tonometry may not be the most appropriate option. For example, device performance could be adversely impacted by alternations in the sympathetic response or impaired perfusion at the peripheral tissue, such as during treatment with adrenergic system modulators (e.g., alpha-adrenergic antagonists) (Zou et al., 2010) and in individuals with clinically relevant peripheral vascular disease. Thus, although alternative approaches to sleep apnea assessment might be more appropriate in these groups, use of peripheral artery tonometry-based devices to address the unmet need for better approaches to OSA diagnosis for the majority of individuals would allow in-demand sleep laboratory services to be prioritized for more complex individuals (Fietze et al., 2022).

### 4.1. Looking to the (not too distant) future

Sleep medicine is a rapidly developing field, but the prevalence of OSA is growing and the number of sleep specialists is inadequate to meet the increasing need. This highlights the need for initiatives such as new tools and telehealth to provide safe, effective clinical care to an expanding group of patients (O'Donnell et al., 2020). The move toward greater utilization of telemedicine solutions was accelerated during the COVID-19 pandemic due to lockdowns and social distancing requirements (Monaghesh and Hajizadeh, 2020). It makes sense to capitalize on this momentum to improve the diagnosis and management of SDB, and simple, wearable devices based on measurement of peripheral arterial tone, such as NightOwl^®^ and WatchPAT^®^, can make an important contribution to this. For instance, peripheral arterial tone-based HSATs can provide primary care professionals with easy tools to diagnose OSA. These cloud-based multiple-night HSAT technologies can be beneficial for communities without major medical center for SDB management thus promoting equitable SDB identification, diagnosis, and treatment (Gueye-Ndiaye et al., 2023). Moreover, the technologies provide the possibility of OSA screening in large populations and enable new approaches for a simplified and automated OSA diagnostic procedure and treatment follow-up. HSATs, wearable technologies and advances in telemedicine may also help to strengthen inter-departmental collaboration, thus improving the overall care of patients with OSA (Mahoney, 2020; McNicholas and Pevernagie, 2022).

Overall, the possibility of integrating diagnostic, device therapy and patient clinical data is attractive, and facilitates a more holistic approach to patient management. New-generation wearable devices that record a variety of signals to provide information on sleep stage/quality, arousals, sleep position, and a variety of SDB metrics (such as hypoxic burden) (Trzepizur et al., 2022) will provide a more complete picture to inform clinical decision making throughout the patient journey. Better understanding of patient phenotypes will allow specific characteristics to be linked to treatment outcomes (Mazzotti et al., 2019). The variety of accurate data obtained from new connected devices could be used to inform both diagnostics and clinical decision making based on sleep-related breathing parameters, age, symptoms and risks (Hajipour et al., 2023), and in accordance with current clinical recommendations and guidelines (Patil et al., 2019; Randerath et al., 2021; Grote et al., 2023). The new capabilities provided by new technologies and innovations bring new capabilities, such as use of the same device to diagnose sleep apnea and then monitor therapy compliance and efficacy. For example, one currently available peripheral artery tonometry-based device (NightOwl^®^) has 10 nights of battery capacity. The ability to record over 10 consecutive nights could allow three nights for diagnostic studies (AHI, oxygen desaturation index, hypoxic burden and patient-reported outcome measures) followed by sevn nights to implement and monitor a personalized treatment plan, including assessment of changes in hypoxic burden and patient-reported outcomes (Figure 1). This could contribute to reducing the underdiagnosis of OSA, accelerating access to appropriate treatment, and optimization of OSA therapy.

## Author contributions

DZ: Conceptualization, Writing—original draft, Writing—review and editing. SV: Conceptualization, Writing—original draft, Writing—review and editing. CE: Writing—original draft, Writing—review and editing. DE-T: Writing—original draft, Writing—review and editing, Conceptualization. FL: Conceptualization, Writing—original draft, Writing—review and editing. MA: Conceptualization, Writing—original draft, Writing—review and editing. IF: Writing—original draft, Writing—review and editing.
